# Microstructural characterization data for nuclear graphite samples generated during tribology testing in argon

**DOI:** 10.1016/j.dib.2022.108796

**Published:** 2022-11-29

**Authors:** L. Vergari, J. Quincey, G. Meric de Bellefon, R.O. Scarlat

**Affiliations:** a Department of Nuclear Engineering, University of California, Berkeley (CA); b Kairos Power LLC, Alameda (CA)

**Keywords:** Nuclear graphite, Wear, Friction, Raman spectroscopy, XRD analysis, Microstructural characterization, Tribo-film

## Abstract

This article contains microstructural characterization data related to the research article ‘Self-lubrication of Nuclear Graphite in Argon at High Temperature’, published in *Tribology International.* Digital, optical, and scanning electron micrographs are collected on the pebble and disk samples generated in the tribological tests and in their corresponding reference samples. Surface roughness measurements of the test and control samples are performed using the 3D-depth composition feature of the digital microscope. X-ray diffraction and Raman spectra of the test and reference samples are acquired and peak-fitted according to published literature on nuclear graphite characterization. Plots of the peak-fitted spectra are included in this article; the full list of peak-fitting parameters is provided in the linked data repository.

**Specifications Table**

**Table:** 

Subject	Material Characterization
Specific subject area	Microstructural characterization via microscopy, Raman spectroscopy, and X-Ray diffraction of nuclear graphite samples generated in tribology tests conducted at room temperature and 600°C in controlled argon atmosphere.
Type of data	Table Image Graph Figure
How the data were acquired	Raman spectroscopy data acquired with a Horiba LabRam HR confocal Raman microscope. XRD data acquired with a Rigaku MiniFlex 6G diffractometer. Digital microscopies are acquired with a Keyence VHX-6000 digital microscope with automatic stage for 3D measurements. Optical micrographs are acquired with a Nikon Eclipse LV150 NL microscope. SEM micrographs are acquired with a Scios 2 dual beam SEM/FIB.
Data format	Raw images: Digital, optical, SEM micrographs Annotated images: Digital, optical, SEM micrographs, Surface roughness images. Raw data: XRD and Raman data Analyzed data: Peak fitting of XRD and Raman data. Microstructural parameters estimated from XRD and Raman.
Description of data collection>	Optical and digital micrographs , SEM micrographs, XRD patterns and Raman spectra acquired in air at room temperature.
Data source location	XRD patterns and digital micrographs collected at Kairos Power. Optical micrographs and SEM micrographs collected at UC Berkeley. Raman spectra collected at the Joint Center for Artificial Photosynthesis at the Lawrence Berkeley National Lab.
Data accessibility	Repository name: mendeley.com Data identification number: DOI: 10.17632/n8w8w3fhhp.3 Research data: https://data.mendeley.com/datasets/n8w8w3fhhp
Related research article	Vergari, L., J. Quincey, G. Meric de Bellefon, T. Merriman, M. Hackett, and R. O. Scarlat. "Self-lubrication of nuclear graphite in argon at high temperature." Tribology International 177 (2023): 107946. https://doi.org/10.1016/j.triboint.2022.107946

## Value of the Data

• This article provides microstructural characterization data of nuclear graphite samples generated in tribology tests.
• The data included in the article are useful to elucidate the wear and lubrication mechanisms occurring in graphite-graphite sliding in inert environment.
• The data highlights the difference in microstructure after room temperature and high temperature tests.
• The data are beneficial to engineers interested in quantifying wear rates of graphite pebbles used in nuclear reactors and to the materials scientists and engineers interested in graphite friction and wear mechanisms.
• The characterization data can be used as validation data for computational models of graphite wear in argon.

## 1 Objectives

This article contains raw and analyzed characterization data for graphite samples generated in tribology testing. Microstructural characterization of tribology samples is necessary to identify wear mechanisms and extent of wear. This data article augments the companion research article [1] by (i) providing raw and analyzed XRD data, not included in the research article, (ii) supplementing the micrographs in the research article with additional optical, digital, and electron images, and (iii) showing Raman peak-fitting parameters for all the classes of samples generated in the tests.

## 2 Data description

Table 1 lists the samples for which characterization data are available in this manuscript. Section 2.1 contains micrographs of the samples (grouped by type of technique). Section 2.2 contains Raman data, including raw spectra and peak-fitting parameters. Section 2.3 contains XRD data.

**Table 1:** Sample Matrix for microstructural characterization.

Sample Name	Description	Testing Temperature (°C)
Pebble Reference	Non-worn surface on graphite pebble	-
Disk Reference	Non-worn surface on graphite disk	-
WS1	Wear spots on graphite pebble	21
WS4		601
WT1	Wear tracks on graphite disk	21
WT4		601

### 2.1 Microscopy

**Fig. 1:**
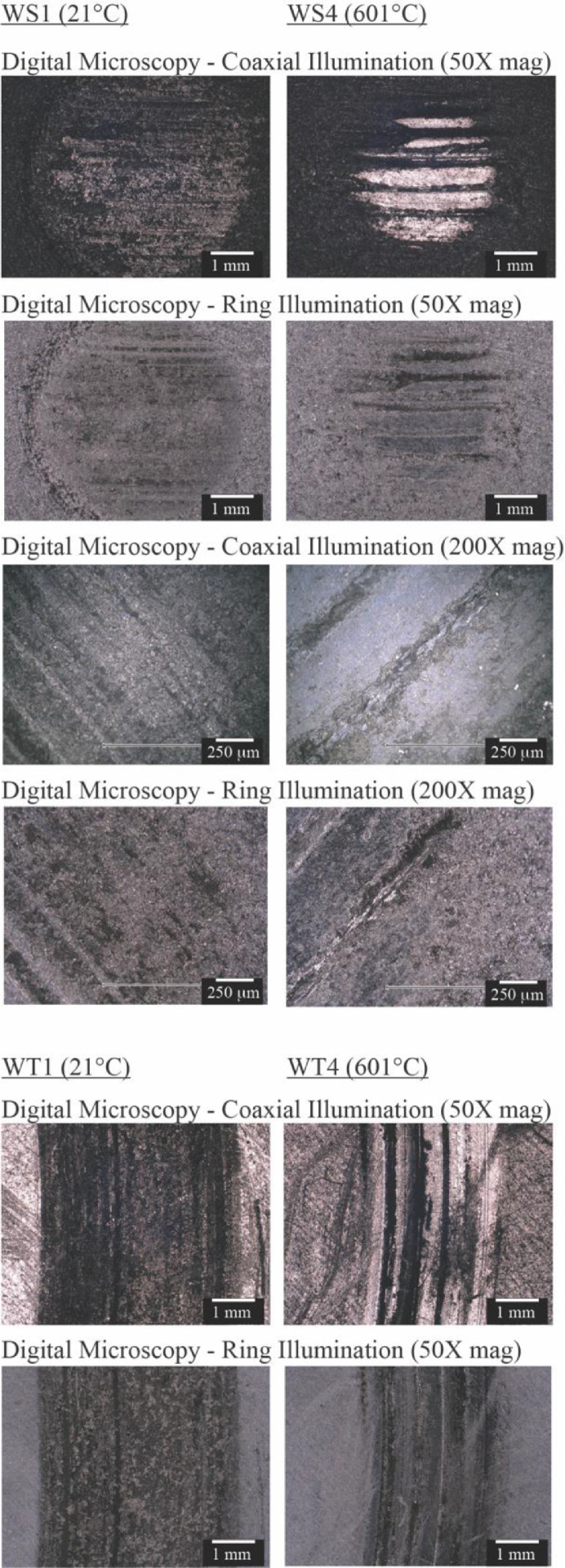
Digital microscopies under coaxial and ring illumination of the wear spots and wear tracks.

Fig. 1 shows the digital micrographs of wear spots and wear tracks. Microscopies are collected using coaxial illumination and ring illumination. Wear spots are imaged at two levels of magnification (50X and 200X). Wear tracks are imaged at 50X magnification. The images under coaxial light clearly illustrate the presence of two regions: a smooth region, which appears bright, and a rough region, which appears dark. The distinction between the two is less evident under ring illumination. Coaxial illumination micrographs of the reference pebble and disk surface are included in the companion article [1].

**Fig. 2:**
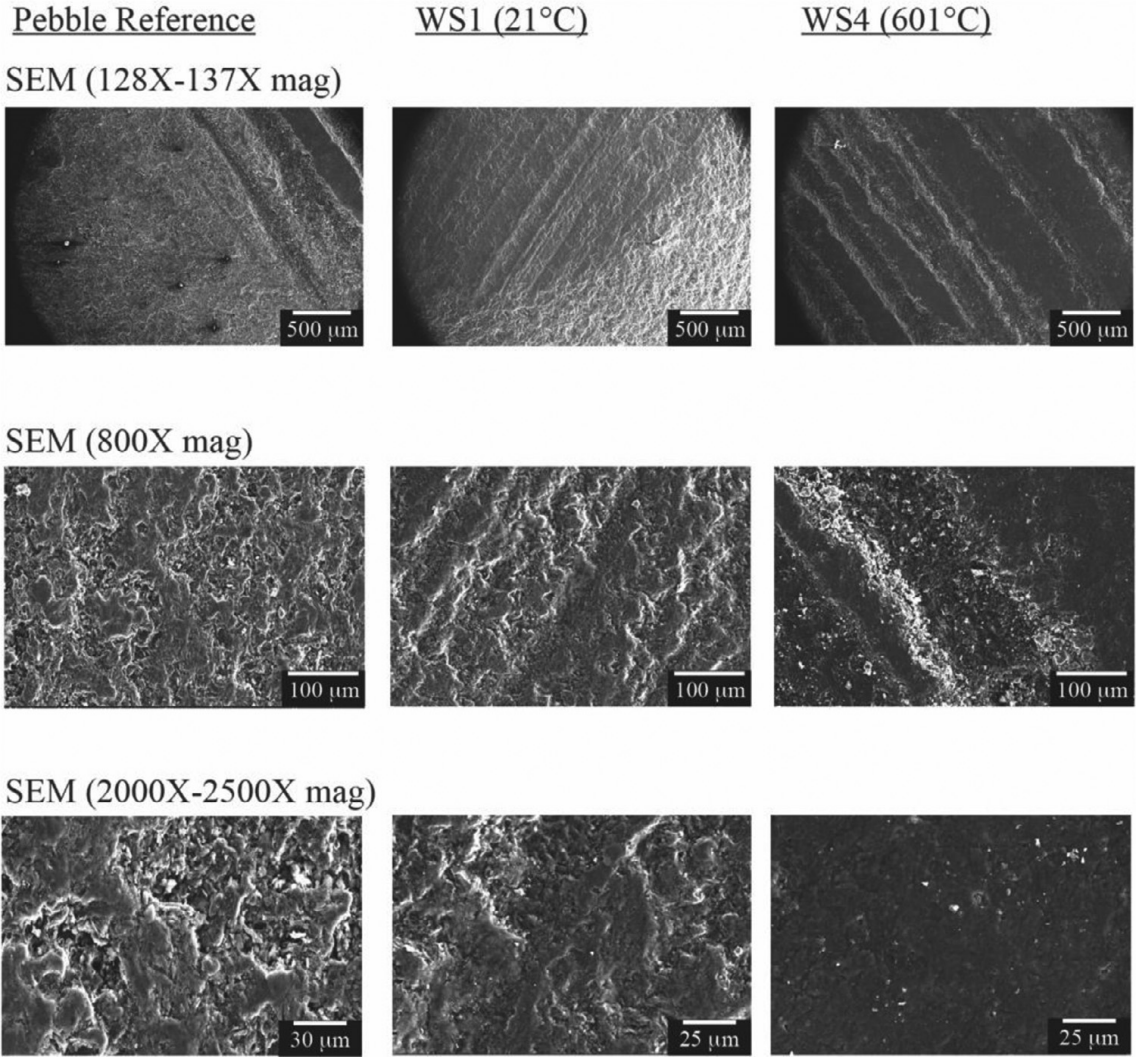
SEM images of the pebble reference surface and the wear spots.

Fig. 2 shows the SEM micrographs for the wear spot and the reference pebble. Images are shown at three different levels of magnification and illustrate the texture of worn samples and the reference surface.

**Fig. 3:**
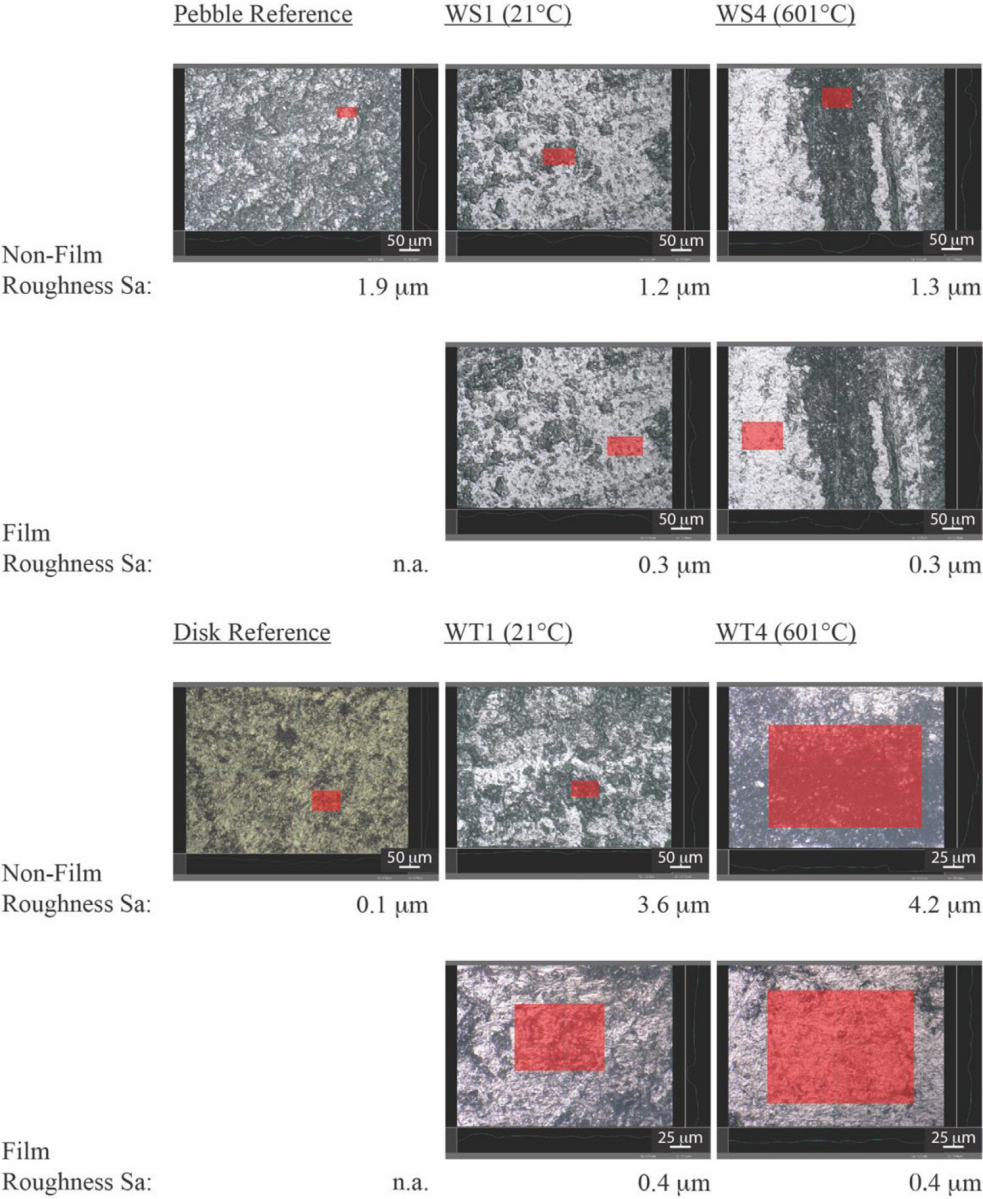
Sa roughness on the reference samples and on the wear spot and wear tracks. Roughness is measured on the areas shaded in red.

Fig. 3 shows the snapshots from the Keyence VHX Software corresponding to the roughness measurements. Roughness is measured using the 3D-Depth Composition module of the microscope. Micrographs are acquired using 500X-1000X magnification and roughness is measured on an area of approximately 2000-10000 µm^2^. Fig. 3 reports the surface averaged roughness Sa for the areas annotated in red and highlights the difference in roughness between the film and the non-film regions.

**Fig. 4:**
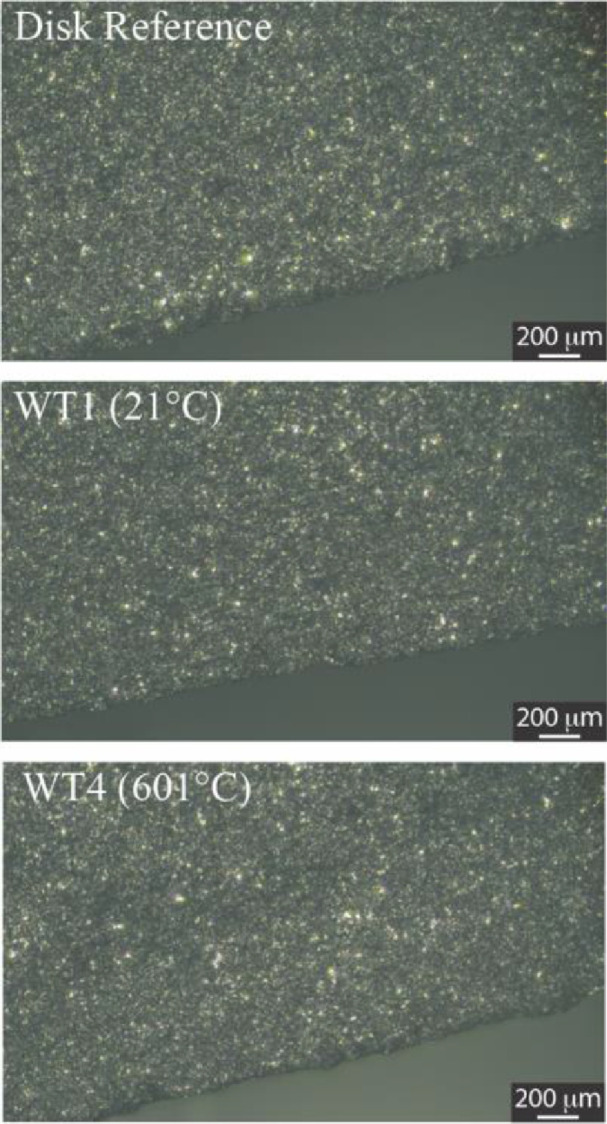
Optical micrographs of the cross-sections of the reference disks and of the wear tracks generated at room temperature and high temperature.

Fig. 4 includes the optical micrographs (50X magnification) of the cross-sections of the wear tracks and of the reference disk. The micrographs do not show signs of deep damage due to wear at either temperature.

### 2.2 Raman spectroscopy

**Fig. 5:**
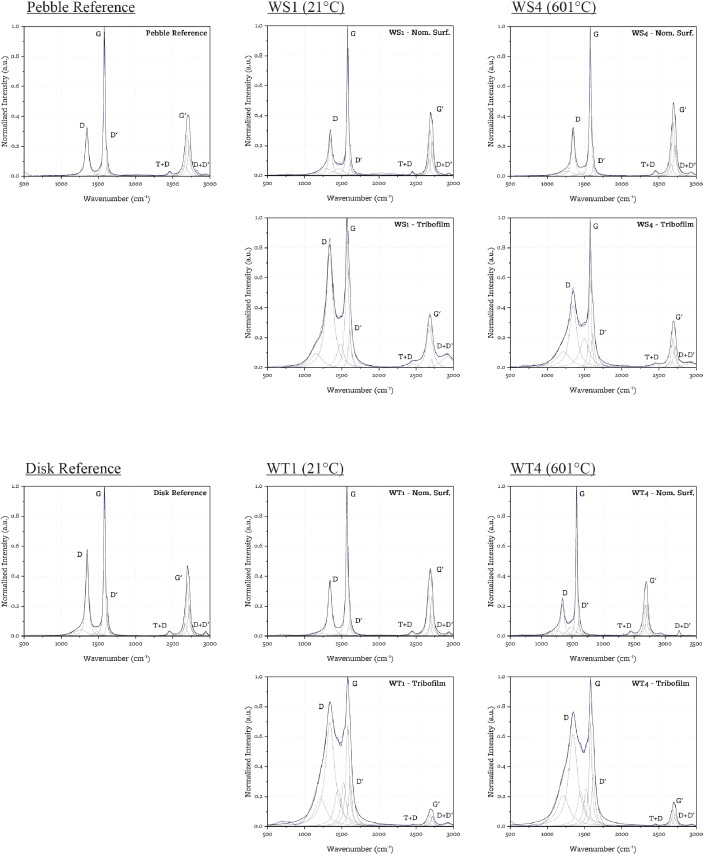
Peak-fitted Raman spectra for the reference samples, the wear spots, and the wear tracks. Original data in solid blue, individual peaks in dashed black, reconstructed spectrum in solid black.

Raman spectra are acquired on 10 points of reference samples, 20 points on each wear spot (10 nominal surface, 10 tribo-film), and on 10 points of each wear track (5 nominal surface, 5 tribo-film). Each Raman spectrum is fitted with 10 Lorenzian peaks. The peak parameters of all spectra are included in the linked data repository. Fig. 5 shows the peak fitted Raman spectra for one point on each reference sample and two points on each wear spot and wear track (one in point in film region, one point in non-film region).

From the Raman spectra, the crystallite dimension in the basal direction La is estimated through the empirical correlations in Eq. (1) [2] and Eq. (2) [3].

(1) $$L_{a}\left(nm\right)=2.4\;10^{-10}\lambda_{l}^{4}\left(\frac{A_{G}}{A_{D}}\right)$$

(2) $$L_{a}\left(nm\right)=\frac{430}{FWHM_{G}-14}$$

Where $\lambda_{l}$ is the laser wavelength, A_x_ and FWHM_x_ are the area (in arbitrary units) and full width at half maximum (in cm^−1^) of the *x* band (*x*=G, D, G’, etc.).

The crystallite dimension in the axial direction (Lc) is estimated with Eq. (3), developed by [4]:

(3) $$L_{c}\left(nm\right)=10+\frac{10}{1.05-R}$$

Where R is the degree of stacking order (i.e. the volume fraction of graphitized regions), which can be estimated from the peak-fitting parameters of the G’ band [4]:

(4) $$R=\frac{\alpha_{g}}{\alpha_{g}+\alpha_{t}}=\frac{A_{G^{\prime }3DB}}{A_{G^{\prime }3DB}+A_{G^{\prime }2D}}$$

Where $\alpha_{g}$ and $\alpha_{t}$ are the volume fractions of the graphitized and turbostratic regions.

Table 2 reports the average microstructural parameters and their standard deviations for the different samples. The full set of microstructural parameters is included in the linked data repository spreadsheet.

**Table 2:** Microstructural parameters estimated via Raman spectroscopy.

	Reference		21°C tests				601°C tests			
Test Sample	Pebble Ref	Disk Ref	Pebble: WS1		Disk: WT1		Pebble: WS4		Disk: WT4	
Location on sample (N=number of sampled points)	Nominal Surface (N=10)	Nominal Surface (N=10)	Nominal Surface (N=10)	Tribo-film (N=10)	Nominal Surface (N=5)	Tribo-film (N=5)	Nominal Surface (N=10)	Tribo-film (N=10)	Nominal Surface (N=5)	Tribo-film (N=5)

La (nm) [2]*from D/G Area ratios*	25.6 ±4.6	18.0 ±2.3	20.5±7.0	10.7 ±2.3	16.2 ±8.3	9.0 ±2.2	31.1 ±6.3	11.2 ±2.3	23.0 ±7.0	8.0 ±1.8

La (nm) [3]*from FWHM(G)*	12.6 ±1.5	13.5 ±1.1	11.7±1.4	5.4 ±3.4	6.6 ±3.2	4.4 ±1.2	10.6 ±1.9	5.3 ±1.4	7.6 ±1.1	4.0 ±1.0

Lc (nm) [4]*from G’ peak decomposition in G’2D and G’3DB*	24.2 ±1.1	24.5 ±0.9	25.3±1.2	23.3 ±1.9	25.3 ±3.0	26.0 ±2.3	25.4 ±1.6	24.4 ±2.0	25.5 ±1.2	26.9 ±0.8

### 2.3 XRD

X-ray diffraction (XRD) spectra using cobalt Kα radiation on a Rigaku MiniFlex 6G diffractometer are acquired on the reference samples, on the wear spots and on the wear tracks. Fig. 5 shows the collected diffraction patterns. The diffraction patterns are peak fitted using Voigt functions using Rigaku SmartLab Studio II. Silicon powder (NIST SRM 640e) is used as standard material for peak locations and FWHM. The peak parameters of the silicon standard and the graphite samples are included in the linked data repository. From the diffraction pattern, the lattice parameters *a* and *c*, the degree of graphitization *g*, the crystallite sizes Lc and La can be estimated from the location and the width of the (002), (004) and (110) peaks, as described in [5] and previously performed for nuclear graphite in [6,7].

Table 3 collects the crystallite parameters obtained by fitting the XRD patterns.

**Fig. 6:**
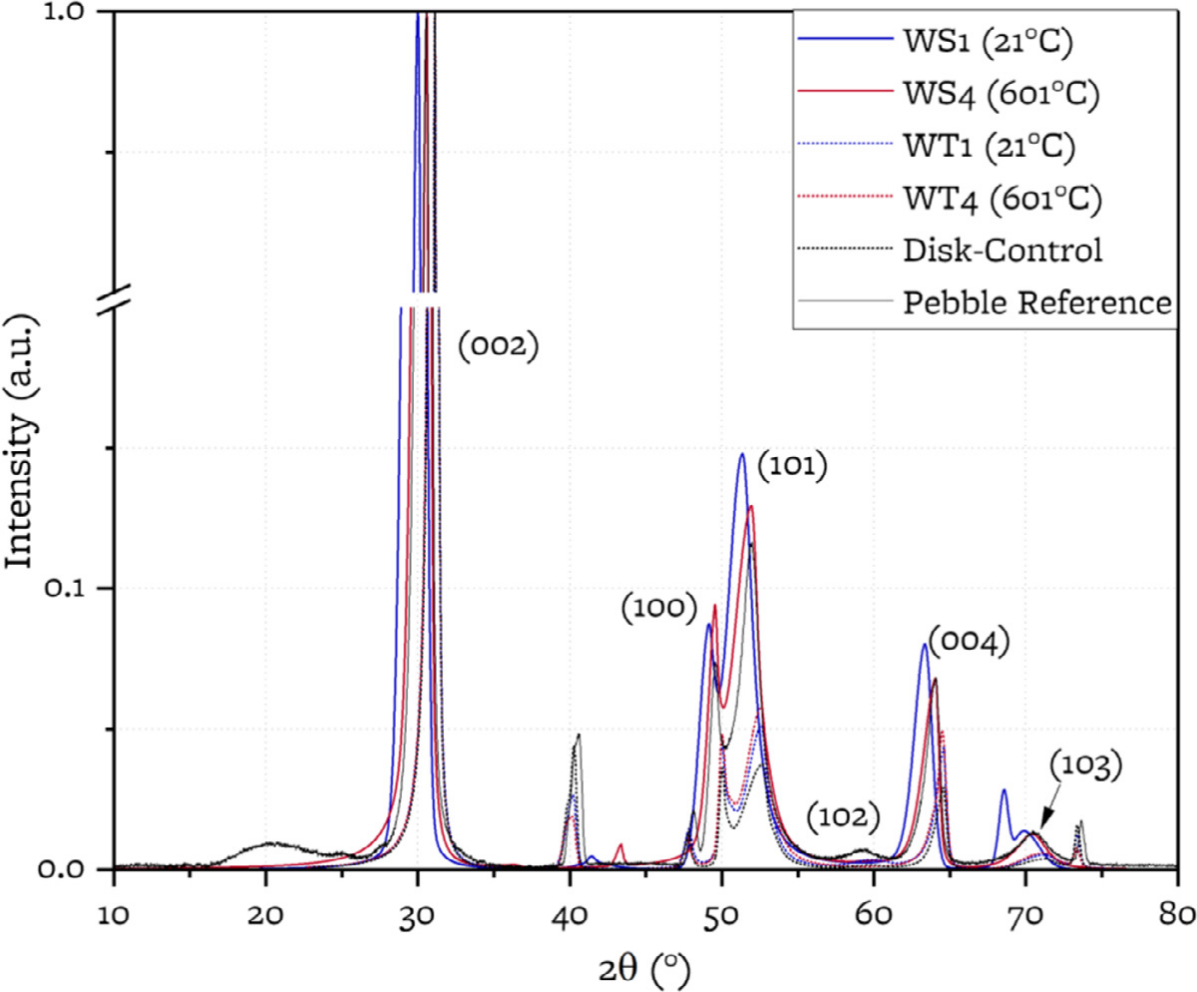
XRD patterns of wear spots (solid lines, WS1, WS4), wear tracks (dashed lines, WT1, WT4) and reference samples. (Pebble Reference solid black, Disk Reference dashed black). Patterns pertaining to samples tested at 21°C shown in blue, patterns of samples tested at 601°C shown in red.

**Table 3:** Microstructural parameters obtained by XRD.

	Reference		21°C test		601°C test	
Test Sample	Pebble Reference	Disk Reference	Pebble: WS1	Disk: WT1	Pebble: WS4	Disk: WT4
a (nm)	0.247	0.246	0.250	0.244	0.248	0.247
c (nm)	0.673	0.673	0.676	0.673	0.673	0.673
g	87%	90%	69%	90%	88%	90%
La (nm)	14	12	11	12	11	12
Lc (nm)	12	22	9	21	12	19

## 3 Experimental Design, Materials and Methods

### 3.1 Materials

The tribology experiments are performed using ET-10 nuclear graphite provided by Ibiden. The samples are manufactured in the form of 4 cm diameter spheres and 10 cm diameter disks. Prior to the tribological tests, the samples are cleaned with acetone in an ultrasonic bath and baked in Argon atmosphere at 600°C for 4 hours.

### 3.2 Characterization Methods

Digital micrographs and profilometry are collected using a Keyence digital microscope VHX-6000 with brightfield coaxial illumination. The microscope has an automated stage and a 3D-Depth Composition module that allow for 3D image acquisition and roughness measurements. Optical micrographs are collected using a Nikon Eclipse LV150NL microscope. SEM micrographs are collected with a Scios 2 dual beam SEM/FIB. X-ray diffraction (XRD) is performed using a Rigaku MiniFlex 6G diffractometer (150 mm goniometer radius), using Co radiation (Kα=0.179 nm). A divergence slit width of 1.25° is used. Diffraction data are collected in the 2θ range of 10° to 90° with a scanning speed of 2°/min and a step size of 0.01°. To fit the sample holder, the sections of the pebbles containing the wear spots are removed and cut down to cubes of size 1 cm x 0.5 cm x 0.5 cm using a hacksaw and preserving the wear spot or wear track undisturbed as much as achievable. Using cobalt Kα radiation, the attenuation length of the X-ray beam is 800 um (estimated using an exponential attenuation with mass attenuation coefficient of 7 cm^2^/g [8]) and the sampling diameter is in the order of hundreds of microns [7]. Silicon powder (NIST SRM 640e) is used as standard reference material for peak locations and full width at half-maximum. The XRD patterns are background subtracted using a b-spline baseline, normalized, and fitted with Voigt functions using Rigaku SmartLab Studio II.

Raman spectra are collected using a Horiba LabRam HR confocal Raman microscope with a 532 nm laser source and optical magnification of 50x. Upon acquisition, the spectra are background-subtracted using an 8-degree polynomial baseline and normalized. For the extraction of microstructure parameters, the spectra are fitted using Lorentzian functions on OriginPro 2021b.

## Ethics Statement

The data presented in this articles are generated from research activities that did not involve human subjects, animal experiments, or social media platforms.

## CRediT authorship contribution statement

**L. Vergari:** Conceptualization, Methodology, Formal analysis, Investigation, Data curation, Writing – original draft, Visualization, Project administration. **J. Quincey:** Formal analysis, Investigation, Data curation, Writing – review & editing. **G. Meric de Bellefon:** Conceptualization, Writing – review & editing, Resources, Supervision, Project administration. **R.O. Scarlat:** Conceptualization, Writing – review & editing, Resources, Supervision, Project administration.

## Declaration of Competing Interest

The authors declare that they have no known competing financial interests or personal relationships that could have appeared to influence the work reported in this paper.

The authors declare the following financial interests/personal relationships which may be considered as potential competing interests:

At the time at which the article is published, L. Vergari, J. Quincey, G. Meric de Bellefon, and R.O. Scarlat have interests in or relationships with entities that are commercializing molten salt technology. The content of this manuscript or the direction of the research presented herein was not influenced by these entities, nor by the author's relationships with these entities.
